# An Observational, Prospective, Multicenter, Registry-Based Cohort Study Comparing Conservative and Medical Management for Patent Ductus Arteriosus

**DOI:** 10.3389/fped.2020.00434

**Published:** 2020-07-31

**Authors:** Emel Okulu, Omer Erdeve, Zehra Arslan, Nihal Demirel, Huseyin Kaya, Ismail Kursad Gokce, Sabahattin Ertugrul, Merih Cetinkaya, Gokhan Buyukkale, Ferda Ozlu, Huseyin Simsek, Yalcin Celik, Hilal Ozkan, Nilgun Köksal, Baris Akcan, Munevver Turkmen, Kiymet Celik, Didem Armangil, Ali Bulbul, Kadir Serafettin Tekgunduz, Mehmet Yekta Oncel, Funda Tuzun, Ebru Ergenekon, Hacer Ergin, Saadet Arsan, Turkish Neonatal Society INTERPDA Study Group

**Affiliations:** ^1^Division of Neonatology, Department of Pediatrics, Ankara University School of Medicine, Ankara, Turkey; ^2^Department of Neonatology, Etlik Zubeyde Hanim Women's Health Teaching and Research Hospital, University of Health Sciences, Ankara, Turkey; ^3^Division of Neonatology, Department of Pediatrics, Ankara Yildirim Beyazit University School of Medicine, Ankara, Turkey; ^4^Division of Neonatology, Department of Pediatrics, Inonu University School of Medicine, Malatya, Turkey; ^5^Division of Neonatology, Department of Pediatrics, Dicle University School of Medicine, Diyarbakir, Turkey; ^6^Department of Neonatology, University of Health Sciences, Kanuni Sultan Suleyman Training and Research Hospital, Istanbul, Turkey; ^7^Division of Neonatology, Department of Pediatrics, Cukurova University School of Medicine, Adana, Turkey; ^8^Division of Neonatology, Department of Pediatrics, Mersin University School of Medicine, Mersin, Turkey; ^9^Division of Neonatology, Department of Pediatrics, Uludag University School of Medicine, Bursa, Turkey; ^10^Division of Neonatology, Department of Pediatrics, Adnan Menderes University School of Medicine, Aydin, Turkey; ^11^Neonatal Intensive Care Unit, Diyarbakir Gazi Yasargil Training and Research Hospital, Diyarbakir, Turkey; ^12^Neonatal Intensive Care Unit, Koru Hospital, Ankara, Turkey; ^13^Department of Neonatology, University of Health Sciences, Sisli Etfal Hamidiye Training and Research Hospital, Istanbul, Turkey; ^14^Division of Neonatology, Department of Pediatrics, Ataturk University School of Medicine, Erzurum, Turkey; ^15^Division of Neonatology, Department of Pediatrics, Izmir Katip Celebi University School of Medicine, Izmir, Turkey; ^16^Division of Neonatology, Department of Pediatrics, Dokuz Eylul University School of Medicine, Izmir, Turkey; ^17^Division of Neonatology, Department of Pediatrics, Gazi University School of Medicine, Ankara, Turkey; ^18^Division of Neonatology, Department of Pediatrics, Pamukkale University School of Medicine, Denizli, Turkey

**Keywords:** patent ductus arteriosus, preterm, conservative, management, morbidity, mortality, ibuprofen, paracetamol

## Abstract

No consensus has been reached on which patent ductus arteriosus (PDAs) in preterm infants require treatment and if so, how, and when they should be treated. A prospective, multicenter, cohort study was conducted to compare the effects of conservative approaches and medical treatment options on ductal closure at discharge, surgical ligation, prematurity-related morbidities, and mortality. Infants between 24^0/7^ and 28^6/7^ weeks of gestation from 24 neonatal intensive care units were enrolled. Data on PDA management and patients' clinical characteristics were recorded prospectively. Patients with moderate-to-large PDA were compared. Among the 1,193 enrolled infants (26.7 ± 1.4 weeks and 926 ± 243 g), 649 (54%) had no or small PDA, whereas 544 (46%) had moderate-to-large PDA. One hundred thirty (24%) infants with moderate-to-large PDA were managed conservatively, in contrast to 414 (76%) who received medical treatment. Eighty (62%) of 130 infants who were managed conservatively did not receive any rescue treatment and the PDA closure rate was 53% at discharge. There were no differences in the rates of late-onset sepsis, necrotizing enterocolitis (NEC), retinopathy of prematurity, intraventricular hemorrhage (≥Grade 3), surgical ligation, and presence of PDA at discharge between conservatively-managed and medically-treated infants (*p* > 0.05). Multivariate analysis including perinatal factors showed that medical treatment was associated with increased risk for mortality (OR 1.68, 95% Cl 1.01–2.80, *p* = 0.046), but decreased risk for BPD or death (BPD/death) (OR 0.59, 95%Cl 0.37–0.92, *p* = 0.022). The preferred treatment options were ibuprofen (intravenous 36%, oral 31%), and paracetamol (intravenous 26%, oral 7%). Infants who were treated with oral paracetamol had higher rates of NEC and mortality in comparison to other treatment options. Infants treated before postnatal day 7 had higher rates of mortality and BPD/death than infants who were conservatively managed or treated beyond day 7 (*p* = 0.009 and 0.007, respectively). In preterm infants born at <29 weeks of gestation with moderate-to-large PDA, medical treatment did not show any reduction in the rates of open PDA at discharge, surgical or prematurity-related secondary outcomes. In addition to the high incidence of spontaneous closure of PDA in the first week of life, early treatment (<7 days) was associated with higher rates of mortality and BPD/death.

## Introduction

Patent ductus arteriosus (PDA) is the most common cardiovascular condition in preterm infants. Although the ductus arteriosus (DA) is closed spontaneously in most of the infants born at >28 weeks of gestation (i.e., at the end of the first week of life), persistent patency of the DA is observed in 50–70% of infants born at <28 weeks of gestation, and lasts for weeks after birth (1, 2).

PDA is associated with mortality and morbidities including necrotizing enterocolitis (NEC), pulmonary hemorrhage, intraventricular hemorrhage (IVH), retinopathy of prematurity (ROP), bronchopulmonary dysplasia (BPD), and poor neurodevelopmental outcomes. These morbidities are caused by the left-to-right shunt through the DA that may result in pulmonary hyperperfusion and systemic hypoperfusion (3–6). This association has encouraged neonatologists to treat PDA in order to reduce these associated morbidities, but there remains a wide variety of management options for PDA across centers worldwide (7, 8).

Despite PDA's association with prematurity-related morbidities, no randomized controlled trial to date has demonstrated improvements in BPD, long-term neurodevelopmental outcomes or mortality after medical or surgical treatment of PDA (1, 4, 9–11). Therefore, centers have been performing conservative follow-ups for PDA, especially since higher rates of spontaneous closure rates have been reported in the current era (12, 13). A recent study that compared early routine treatment (ERT) of PDA with conservative management showed that ERT did not reduce either PDA ligations or the presence of a PDA at discharge, and did not improve secondary outcomes, However, ERT was associated with higher rates of late-onset sepsis (LOS) and death in infants born at >26 weeks of gestation (14). Therefore, we aimed to establish a prospective online registry database to examine the variations in PDA management for a nationally-based cohort, and evaluate the effects of PDA management strategies on the rates of PDA closure, PDA ligation, associated morbidities, and survival in preterm infants.

## Materials and Methods

After the establishment of the Experiences in Timing and Choices for Ductal Closure in Patent Ductus Arteriosus (INTERPDA) Study Online Registry in January 2017, a multicenter prospective observational nationally-based cohort study was conducted. Infants who were born at gestational weeks between 24^0/7^ and 28^6/7^ and underwent echocardiography in first 3 weeks of life after birth were included. Clinical directors in neonatal intensive care units (NICUs) nationwide were made aware of the study, and 24 NICUs participated. The NICUs were asked to add all hospitalized patients per day to the registry database using an online standard, patient-specific electronic case report form (eCRF) for a 2-years period. Data were then collected prospectively and registered by trained neonatologists.

The study was approved by the Online Studies Scientific Steering Committee of the Turkish Neonatal Society and by the Institutional Review Board of Ankara University. Written informed consent was obtained from the parents or guardians of the newborns. The study was registered at ClinicalTrials.gov (NCT02910609).

The eCRF included demographic and clinical findings including gestational age, birth weight, gender, type of delivery, the presence of prolonged premature rupture of membranes (PPROM) which was defined as rupture of membranes >18 h before labor and before 37 weeks' gestation, antepartum preeclampsia (15); clinical chorioamnionitis, the use of any antenatal steroid, respiratory distress syndrome (RDS) (16), clinical or culture proven early-onset sepsis in addition to echocardiographic assessment results, type of PDA, management provided to the infants (conservative or medical), the timing of any medication used, type of preferred drug, outcomes of management on ductal closure at discharge, surgical ligation, and adverse events. Associated morbidities such as culture proven LOS, NEC (17), IVH (18), ROP (19), BPD (20), and mortality were also recorded.

### Echocardiographic Assessment

Diagnosis of PDA was based on clinical and/or echocardiographic studies, which included 2-dimensional imaging, M-mode, color flow mapping, and Doppler interrogation. A moderate-to-large PDA was defined as an internal ductus diameter >1.5 mm, left atrium-to-aortic root ratio >1.5, and reverse diastolic flow in the descending aorta. A ductus that failed to meet these criteria was defined as small PDA.

### Management of PDA

The decision for the management of PDA was left up to each center according to their standard practices.

Infants with moderate-to-large PDA were grouped according to their PDA management as conservative approach or medical treatment. Patients' demographic and clinical findings, outcomes of preferred PDA management, rates of associated morbidities, mortality rate, and BPD or death (BPD/death) were compared between the two groups.

### Statistical Analysis

Frequency and percentage (*n*, %) were used to describe categorical data. Pearson Chi-squared test was used to assess the relationship between categorical variables when the test requirements were met. Otherwise, Fisher's exact test or Fisher-Freeman-Halton Exact test was used to test independence between categorical variables depending on the table size. For numerical dependent variables Mann-Whitney *U*-test was used for comparisons between groups due to the fact that the parametric test assumptions were not met. Normality of numerical variables was assessed by Kolmogorov-Smirnov test. Homogeneity of group variances were evaluated by Levene test. Descriptive statistics were reported as minimum value, maximum value, median (interquartile range-IQR). In addition, mean ± standard deviation was also given. After conducting univariate analysis to estimate effects of managements for PDA on mortality and BPD/death by using generalized estimating equations (GEE); multivariate models were evaluated with QICC criteria. All regression analysis was based on GEE, we used non-robust standard errors and accounted for the clustering of infants within center (21). Odds ratio are presented with 95% Cl. Statistical analysis was performed using the IBMM SPSS Statistics version 23 for Windows.

## Results

### Demographic Data of the Study Group

**Figure 1:**
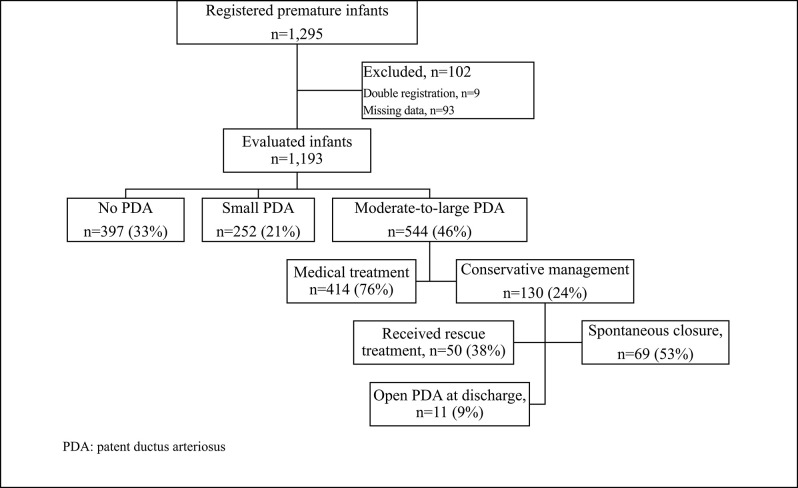
The flow chart of the study.

The final study sample included 1,193 infants after exclusion of 102 from a total of 1,295 because of double-record or missing data (Figure 1). The mean gestational age and birth weight of infants were 26.7 ± 1.4 weeks and 926 ± 243 g, respectively. Of the 1,193, 523 (44%) infants were born at <27 weeks' gestation, and 730 (62%) weighed <1,000 g. The prenatal and neonatal demographic characteristics of the infants enrolled in the study are displayed in Table 1.

**Table 1:** The demographic data of the infants included in INTERPDA study.

	**No. of patients (*n* = 1,193)**
Gestational age (wk)^*^	26.7 ± 1.4
Birth weight (g)^*^	926 ± 243
Gender (male), *n* (%)	605 (51)
Type of delivery (C/S), *n* (%)	949 (79)
Multiple gestation, *n* (%)	231 (19)
PPROM, *n* (%)	288 (24)
Preeclampsia, *n* (%)	305 (26)
Chorioamnionitis, *n* (%)	112 (9)
Antenatal steroid, *n* (%)	83 (70)
Delivery room intubation, *n* (%)	555 (46)
Surfactant use, *n* (%)	956 (80)
RDS, *n* (%)	986 (83)
Early-onset sepsis, *n* (%)	436 (36)

*C/S, cesarean section; PPROM, prolonged premature rupture of membranes; RDS, respiratory distress syndrome*.

* *Data given mean ± SD*.

The median of the echocardiographic assessment time was 3 days (interquartile range, IQR = 2 days). The incidence of small PDA and no PDA was 252 (21%) and 397 (33%), respectively; whereas 544 (46%) patients had moderate-to-large PDA on echocardiographic evaluation. The incidence of moderate-to-large PDA was 63, 60, 45, 40, and 38% according to a gestational age of 24, 25, 26, 27, and 28 weeks, respectively.

### Data of Infants With Moderate-to-Large PDA

One hundred thirty (24%) infants with moderate-to-large PDA were managed conservatively, in contrast to 414 (76%) infants who received medical treatment. The data for these infants are displayed in Table 2. Infants with moderate-to-large PDA in both the conservative and medically-treated groups showed similar gestational age, birth weight, incidences of maternal preeclampsia, chorioamnionitis and PPROM, antenatal steroid use, and early onset sepsis (*p* > 0.05). Higher incidences of delivery room intubation, RDS, and surfactant use were present in medically treated infants (*p* < 0.05).

**Table 2:** Demographic and clinical findings of the conservatively managed and medically treated groups of the infants with moderate-to-large PDA.

	**Conservative**	**Medical**	***P***
	**(*n* = 130)**	**(*n* = 414)**	
Gestational age (w)^*^	26.5 ± 1.4	26.5 ± 1.5	0.89
Birth weight (g)^*^	924 ± 232	877 ± 230	0.06
Gender (male), *n* (%)	65 (30)	202 (49)	0.81
Type of delivery (C/S), *n* (%)	96 (74)	311 (75)	0.77
Multiple gestation, *n* (%)	22 (17)	90 (22)	0.23
PPROM, *n* (%)	40 (31)	94 (23)	0.06
Preeclampsia, *n* (%)	32 (25)	101 (24)	0.96
Chorioamnionitis, *n* (%)	14 (11)	52 (13)	0.58
Antenatal steroid, *n* (%)	88 (68)	288 (70)	0.68
Delivery room intubation, *n* (%)	64 (49)	239 (58)	0.08
Surfactant use, *n* (%)	104 (80)	380 (92)	< 0.001
RDS, *n* (%)	108 (83)	392 (95)	< 0.001
Early-onset sepsis, *n* (%)	48 (37)	166 (40)	0.52
Received respiratory support, *n* (%)	113 (87)	408 (99)	< 0.001
Duration of respiratory support (d)^**^	18 (1–245)	22 (1–181)	0.001
Discharged with open PDA, *n* (%)	25 (19)	80 (19)	0.98
PDA ligation, *n* (%)	8 (6)	29 (7)	0.73
LOS, *n* (%)	71 (55)	216 (52)	0.62
IVH (≥Grade 3), *n* (%)	5 (4)	15 (4)	> 0.05
NEC, *n* (%)	28 (21)	107 (26)	0.32
Moderate-severe BPD^¥^, *n* (%)	25 (47)	93 (49)	0.84
Treated ROP^‡^, *n* (%)	14 (13)	63 (21)	0.06
Mortality, *n* (%)	29 (22)	144 (35)	0.008
BPD/death, *n* (%)	51 (39)	226 (55)	0.002
Duration of hospitalization (d)^**^	59 (2–253)	62 (2–357)	0.79

*BPD, bronchopulmonary dysplasia; C/S, cesarean section; IVH, intraventricular hemorrhage; LOS, late-onset sepsis; NEC, necrotizing enterocolitis; PDA, patent ductus arteriosus; PPROM, prolonged premature rupture of membranes; RDS, respiratory distress syndrome; ROP, retinopathy of prematurity*.

‡ *Infants had at least one eye examination for ROP*.

¥ *Infants survived at PMA 36 weeks' gestation*.

* *Data given mean ± SD*,

** *Data given as median (range)*.

**Figure 2:**
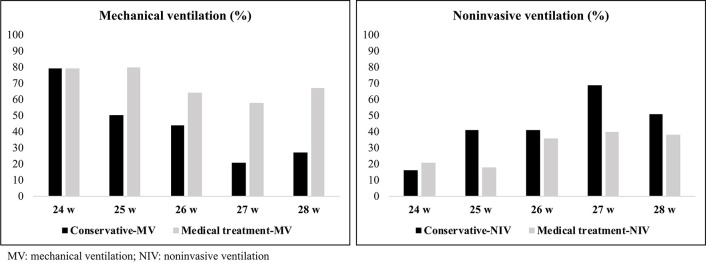
The respiratory support of infants and PDA management.

The respiratory support of infants according to gestational age and PDA management is shown in Figure 2. More infants received any respiratory support in medical treatment group than infants in conservative group (99 vs. 87%, *p* < 0.001). The rate of non-invasive ventilation use as respiratory support was higher in conservatively-managed infants with moderate-to-large PDA (*p* = 0.004), whereas the number of infants receiving mechanical ventilation was higher among medically-treated patients (*p* = 0.011).

There were no differences in the rates of open PDA at discharge, surgical ligation, LOS, NEC (any stage), ROP (received treatment who had at least one eye examination for ROP), IVH (≥Grade 3), BPD [moderate-to-severe who survived at postmenstrual [PMA] 36 weeks of gestation] between the groups (*p* > 0.05), but infants who were medically treated had higher rates of mortality (OR 1.82, 95% Cl 1.15–2.89, *p* = 0.011), and BPD/death (OR 1.81 95% Cl 1.20–2.71, *p* = 0.004) (Table 2). However, multivariate analysis including perinatal factors (gestational age, birth weight, chorioamnionitis, delivery room intubation) showed that medical treatment was associated with increased risk for mortality (OR 1.68, 95% Cl 1.01–2.80, *p* = 0.046), but decreased risk for BPD/death (OR 0.59, 95% Cl 0.37–0.92, *p* = 0.022).

**Figure 3:**
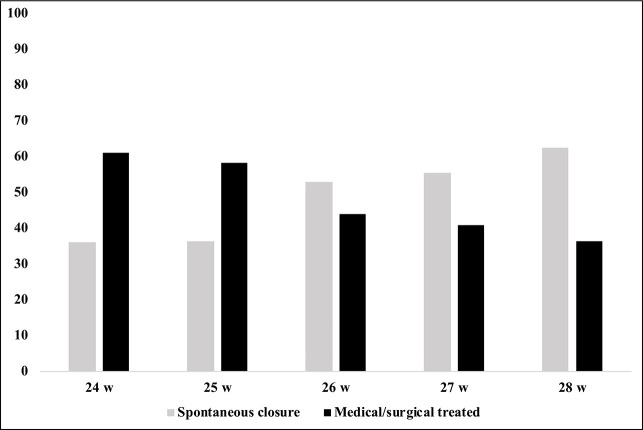
The rates of spontaneous closure or medically/surgical treated ductus according to gestational age.

Eighty (62%) of the 130 infants who were managed conservatively did not receive any rescue treatment during their hospitalization. PDA closure before hospital discharge was observed in 69 infants. The spontaneous closure rate increased with gestational age and reached a peak level of 63% in infants born at 28 weeks of gestation (Figure 3).

We performed a secondary analysis according to gestational age (<27 weeks and ≥27 weeks) to evaluate the effects of PDA management on any of the outcomes (Table 3).

**Table 3:** Outcomes of infants born <27 weeks and ≥27 weeks' gestation.

	**<27 weeks (*****n*** **=** **282)**			**≥27 weeks (*****n*** **=** **262)**		
	**Conservative**	**Medical**	***P***	**Conservative**	**Medical**	***P***
	**(*n* = 68)**	**(*n* = 214)**		**(*n* = 62)**	**(*n* = 200)**	
**DEMOGRAPHIC AND CLINICAL DATA**						
Gestational age (week)^*^	25.3 ± 0.9	25.3 ± 0.8	0.37	27.7 ± 0.5	27.8 ± 0.5	0.29
Birth weight (g)^*^	796 ± 158	754 ± 170	0.06	1,066 ± 219	1,009 ± 212	0.07
Gender (male), *n* (%)	31 (46)	107 (50)	0.52	34 (55)	95 (47)	0.31
Type of delivery (C/S), *n* (%)	47 (69)	146 (68)	0.89	49 (79)	165 (82)	0.53
Multiple gestation, n (%)	12 (18)	42 (20)	0.71	10 (16)	48 (24)	0.19
PPROM, *n* (%)	22 (32)	67 (31)	0.87	18 (29)	27 (13)	0.005
Preeclampsia, *n* (%)	16 (23)	46 (21)	0.72	16 (26)	55 (27)	0.79
Chorioamnionitis, *n* (%)	11 (16)	36 (17)	0.90	3 (5)	16 (8)	0.4
Antenatal steroid, *n* (%)	45 (66)	132 (62)	0.50	43 (69)	156 (78)	0.16
Delivery room intubation, *n* (%)	40 (59)	154 (72)	0.04	24 (39)	85 (42)	0.59
Surfactant use, *n* (%)	61 (90)	206 (96)	0.09	44 (71)	177 (88)	0.001
RDS, *n* (%)	60 (88)	203 (95)	0.05	47 (76)	186 (93)	< 0.001
Early-onset sepsis, *n* (%)	27 (40)	94 (44)	0.54	21 (34)	72 (36)	0.76
Received respiratory support, *n* (%)	61 (90)	213 (99)	< 0.001	52 (84)	195 (97)	< 0.001
Duration of respiratory support (d)^**^	26 (2–245)	23 (1–150)	0.91	14 (1–96)	22 (2–181)	0.001
**OUTCOMES**						
Discharged with open PDA, *n* (%)	16 (23)	53 (25)	0.83	9 (14)	27 (13)	0.83
PDA ligation, *n* (%)	6 (9)	17 (8)	0.81	2 (3)	12 (6)	0.53
LOS, *n* (%)	44 (65)	24 (55)	0.16	27 (43)	98 (49)	0.45
IVH (≥Grade 3), *n* (%)	4 (6)	9 (4)	0.52	1 (2)	6 (3)	> 0.05
NEC, *n* (%)	22 (32)	62 (29)	0.59	6 (10)	45 (22)	0.026
Moderate-severe BPD^¥^, *n* (%)	18 (50)	57 (56)	0.50	11 (50)	49 (47)	0.80
Treated ROP^‡^, *n* (%)	11 (16)	45 (21)	0.38	3 (5)	18 (9)	0.42
Mortality, *n* (%)	24 (35)	111 (52)	0.017	5 (8)	33 (16)	0.09
BPD/death, *n* (%)	37 (54)	150 (70)	0.017	14 (23)	76 (38)	0.025
Duration of hospitalization (d)^**^	71 (2–253)	54.5 (2–357)	0.34	54 (2–120)	63 (3–180)	0.018

*BPD, bronchopulmonary dysplasia; C/S, cesarean section; IVH, intraventricular hemorrhage; LOS, late-onset sepsis; NEC, necrotizing enterocolitis; PDA, patent ductus arteriosus; PPROM, prolonged premature rupture of membranes; RDS, respiratory distress syndrome; ROP, retinopathy of prematurity*.

‡ *Infants had at least one eye examination for ROP*.

¥ *Infants survived at PMA 36 weeks' gestation*.

* *Data given mean ± SD*,

** *Data given as median (range)*.

Despite similar incidences of open PDA at discharge, PDA ligation, and mortality between groups, higher rates of NEC and BPD/death were observed in medically treated infants born at ≥27 weeks of gestation (*p* = 0.026 and *p* = 0.025, respectively). These infants had higher incidences of RDS and surfactant use, and longer duration of respiratory support (median 24 days vs. 12 days, *p* < 0.001) compared to conservatively managed infants. Univariate analysis showed that medical treatment was associated with an increased the risk of NEC and BPD/death (OR 2.7, 95% Cl 1.09–6.68, *p* = 0.031, and OR 2.12, 95% Cl 1.07–4.20, *p* = 0.03, respectively) in infants born at ≥27 weeks of gestation, however multivariate analysis could not demonstrate the similar effect for BPD/death.

The groups did not differ in duration of respiratory support and rate of mechanical ventilation in infants born at <27 weeks of gestation. The rates of open PDA at discharge, surgical ligation, and morbidities did not differ significantly in those who were medically treated and those conservatively managed, but medically treated infants had higher rates of mortality and BPD/death. Univariate analysis indicated odds of increased rates of mortality and BPD/death associated with medical treatment in infants born at <27 weeks of gestation (OR 1.97, 95% Cl 1.12–3.47, *p* = 0.19, and OR 1.96, 95% Cl 1.12–3.44, *p* = 0.19), however these were not significant on multivariate analysis.

### Data Related to Timing and Preferred Medical Treatment Options

The preferred treatment options were ibuprofen (intravenous 36%, oral 31%), and paracetamol (intravenous 26%, oral 7%) as the initial course; indomethacin was not used in any patient. The median starting time of first-course pharmacological treatment was 3 days (IQR = 2.25 days). The timing of medical treatment was not found to be associated with mortality or morbidities. The most common side effects of drug treatment were impairment of renal function and risk of bleeding, which were mostly reported with ibuprofen treatment.

The incidences of open PDA at discharge, surgical ligation, LOS, BPD, and ROP were similar according to the first-course medication selection. However, infants treated with oral paracetamol had higher rates of NEC and mortality, and infants treated with intravenous paracetamol had a higher rate of BPD/death (Table 4).

**Table 4:** Morbidities and mortality according to treatment options.

	**IV ibuprofen**	**Oral ibuprofen**	**IV paracetamol**	**Oral paracetamol**	***p***
	**(*n* = 148)**	**(*n* = 129)**	**(*n* = 108)**	**(*n* = 29)**	
Discharged with open PDA, *n* (%)	28 (19)	19 (15)	24 (22)	9 (31)	0.18
Surgical ligation, *n* (%)	11 (7)	10 (8)	6 (6)	2 (7)	0.91
LOS, *n* (%)	82 (64)	65 (51)	54 (61)	10 (53)	0.08
IVH (≥Gr 3), *n* (%)	3 (2)	6 (5)	5 (5)	1 (3)	0.61
NEC, *n* (%)	40 (27)	32 (25)	20 (18)	15 (52)	0.004
Treated ROP^‡^, *n* (%)	22 (15)	18 (14)	18 (17)	5 (17)	0.93
Moderate-severeBPD^¥^, *n* (%)	36 (44)	39 (59)	28 (58)	3 (30)	0.11
Mortality, *n* (%)	46 (31)	34 (26)	48 (44)	16 (55)	0.002
BPD/death, *n* (%)	75 (51)	63 (49)	71 (66)	17 (59)	0.04

*BPD, bronchopulmonary dysplasia; IVH, intraventricular hemorrhage; LOS, late-onset sepsis; NEC, necrotizing enterocolitis; PDA, patent ductus arteriosus; ROP, retinopathy of prematurity*.

‡ *Infants had at least one eye examination for ROP*.

¥ *Infants survived at PMA 36 weeks' gestation*.

Ninety-six (26%) of the 362 infants received a second course of treatment with ibuprofen (intravenous 34%, oral 35%) and paracetamol (intravenous 23%, oral 8%), and 29 infants received a third course of treatment mostly with intravenous paracetamol (59%).

Infants who received treatment before postnatal day 7 had higher rates of mortality and BPD/death than infants who were conservatively-managed or treated after day 7 (*p* = 0.009 and *p* = 0.007, respectively) (Table 5).

**Table 5:** Data of mortality, surgical ligation and morbidities in infants who were conservatively managed or medically treated infants (<7 days and ≥7 days).

	**Conservative**	**Medical treatment**		***p***
	**(*n* = 130)**	**(*****n*** **=** **414)**		
		**<7 days**	**≥7 days**	
		**(*N* = 363)**	**(*N* = 51)**	
Discharged with open PDA, *n* (%)	25 (19)	72 (20)	8 (16)	0.78
PDA ligation, *n* (%)	8 (6)	24 (7)	5 (10)	0.66
LOS, *n* (%)	71 (55)	188 (52)	28 (55)	0.81
IVH (≥Gr 3), *n* (%)	5 (3.8)	13 (3.6)	2 (3.9)	0.32
NEC, *n* (%)	28 (21)	91 (25)	16 (31)	0.38
Moderate-severe BPD^¥^, *n* (%)	25 (47)	80 (48)	13 (54)	0.83
Treated ROP^‡^, *n* (%)	14 (13)	56 (22)	7 (16)	0.12
Mortality, *n* (%)	29 (22)	131 (36)	13 (25)	0.009
BPD/death, *n* (%)	51 (39)	201 (55)	25 (49)	0.007

*BPD, bronchopulmonary dysplasia; IVH, intraventricular hemorrhage; LOS, late-onset sepsis; NEC, necrotizing enterocolitis; PDA, patent ductus arteriosus; ROP, retinopathy of prematurity*.

‡ *Infants had at least one eye examination for ROP*.

¥ *Infants survived at PMA 36 weeks' gestation*.

## Discussion

In this first nationwide study on the management of PDA in preterm infants born at <29 weeks of gestation, we found that there are management variations between NICUs across Turkey. Although most of the patients with moderate-to-large PDA received medical treatment, there was an increasing trend toward the conservative approach. Medical treatment had no effect on the incidence of surgical ligation or prematurity-related morbidities in the overall study group, and early treatment before day 7 of life was associated with higher mortality and BPD/death. Medical treatment was additionally associated with higher rates of NEC in infants who were born at >27 weeks of gestation.

PDA is present in up to 70% of preterm infants born at <28 weeks of gestation (2, 5). The proportion of very low birth weight (VLBW) infants with PDA was reported to be 36% and 38% in Japan and Canadian networks, respectively (22). According to the Korean Neonatal Network 2017 annual report, 45% of VLBW infants had a PDA (23). In the present study, moderate-to-large PDA was present in 46% of preterm infants born at <29 weeks of gestation and increased to 63% in patients born at 24 weeks of gestation.

Management of PDA in extremely preterm infants remains a topic of debate. Treatment to induce ductal closure has been widely practiced until the last decade, despite lack of evidence that it decreases morbidities and mortality. Meta-analyses of trials using non-steroidal anti-inflammatory drugs have shown effectiveness in accelerating ductal closure, but no reduction in neonatal morbidities regardless of the drugs used, indication, timing, gestational age, or route of administration (1). While medical treatment and surgical ligation can close the ductus, such treatment has had side effects (11, 24). The trend toward conservative treatment has been fueled by recent experience with conservative approaches that demonstrated improved neonatal outcomes and a high rate of spontaneous ductal closure at discharge. Lokku et al. demonstrated that an increasing rate of conservative management of PDA occurred alongside a decrease in medical and/or surgical treatment between 2006 and 2012 in Canada (25). An American cohort study with a large population from 280 NICUs showed a significant decrease in diagnosis and medical/surgical treatment of PDA with no evidence of increased morbidities (26). The recent PDA-TOLERATE trial is the only randomized trial that compared routine treatment of a moderate-to-large PDA present at the end of the first week of life in infants born at <28 weeks of gestation to a conservative approach in reducing neonatal morbidities and/or death. It showed that routine treatment of PDA decreased the incidence of PDA at discharge, but failed to decrease the incidence of BPD, BPD/death, and morbidities. Routine treatment of PDA also appeared to increase the incidence of LOS and death among infants born at >26 weeks of gestation (14). Similarly, the present study's results showed a trend toward the conservative approach in our NICUs across Turkey. In this study, although medical treatment of PDA did not show any reduction in the rates of open PDA at discharge, surgical ligation or neonatal morbidities; it was associated with increased mortality among infants born at <29 weeks of gestation. Medical treatment was preferred in more severe cases in which more mature preterm infants were receiving respiratory support by clinicians. However, subgroup analysis in infants born at <27 weeks of gestation showed that conservative management was not inferior to medical treatment in patients with similar characteristics.

The role played by prolonged PDA exposure in the development of morbidities remains unclear. Studies that compared infants who received indomethacin within the first 3 days of life to infants who received indomethacin only if PDA persisted beyond 7 days with specific rescue criteria showed that infants who received early treatment required less ventilatory and inotropic support and experienced a lower rate of BPD and BPD/death (27–29). Jensen et al. reported that prophylactic-used indomethacin reduced the rates of BPD and BPD/death (30). These studies enrolled and treated infants whose PDA may have been spontaneously closed at the end of the first week. A recent PDA-TOLERATE trial showed that no effect was had on the incidence of BPD if either routine treatment of moderate-to-large PDA was given after the first week or if no treatment was given until severe respiratory or hemodynamic symptoms developed (14). Clyman et al. showed that a significant increase in the incidence of BPD/death required at least 7–13 days of untreated moderate-to-large PDA in infants born at <28 weeks; additional exposure did not add an increase to the incidence of BPD/death (31). In the present cohort study of infants born before 29 weeks of gestation, it was found that early treatment before 7 days of life was associated with higher mortality and BPD/death. Treatment at or after 7 days was not associated with an increase in the risk of incidences of any morbidities. Long-term recent studies demonstrated that a conservative approach, even in the case of prolonged PDA, did not increase the risk of neurodevelopmental impairment (13, 32).

The pharmacological agents used to close the DA are prostaglandin synthase inhibitors such as indomethacin and ibuprofen, or a cyclooxygenase inhibitor like acetaminophen (1, 33–35). Meta-analyses on the efficacy of ibuprofen and indomethacin showed no difference in effectiveness; although ibuprofen can have less intestinal and renal side effects (11). Another meta-analysis that evaluated the association between placebo, indomethacin, ibuprofen, and acetaminophen on PDA closure suggested that a high dose of oral ibuprofen was associated with a higher incidence of PDA closure (36). In some randomized controlled trials, oral paracetamol was found to be as effective and safe as ibuprofen in PDA treatment (37–39). According to the Cochrane review, paracetamol was as effective as ibuprofen without showing any difference in neurodevelopmental outcomes (24). Ibuprofen was the most preferred agent followed by paracetamol (67 vs. 33%) in the present study. The choice of medical agent did not affect the incidence of LOS, BPD, ROP, IVH, surgical ligation, or death. But, unlike the other options, paracetamol was associated with higher rates of NEC, mortality and BPD/death which were not reported in previous studies (38, 39).

The present study had a few limitations. First, it was an observational study. However, to the authors' knowledge, the present report represents one of the largest multicenter studies on the outcomes of persistent PDA. Availability of data from a large national prospective cohort of preterm infants who were admitted to tertiary NICUs strengthened these results. Second, this report details the variations and trends in PDA management between NICUs. The design of the study was such that each center used their individual preferences in the decision of PDA management or treatment selection. Potential of unmeasured markers of early severity of illness and the potential for improper adjustment for each subject's observed time in the study which might be attributable to survivor treatment selection could cause bias, and this should be kept in mind when discussing the effect of treatment on the mortality. Although variable practices of PDA management among centers might have affected the natural course of PDA, this afforded the study the chance to compare the different approaches, agents used, timing of treatment, and PDA outcomes. The authors believe that the results of this large clinical database will lead to further studies to improve uniform guidance for the management of PDA.

## Conclusion

This study provides a summary of the current state of practice for moderate-to-large PDAs in our country, and we think that it will be an important addition to the literature by reporting various treatment and outcome rates. While the majority of NICUs still prefer early symptomatic treatment for moderate-to-large PDA at postnatal 3–5 days of life, there has been a trend toward implementing conservative approaches. As a descriptive study, we observed that medical treatment did not change the incidences of open PDA at discharge, surgical ligation, and morbidities, but was shown to be associated with an increase with mortality. Early medical treatment prior to 7 days of life was not associated with a reduction of the incidences of surgical ligation or morbidities and was associated with higher rates of mortality and BPD/death. Although a conservative approach seems to be preferable option for infants born at <29 weeks of gestation that are suffering from moderate-to-large PDA, we still need well-designed randomized controlled trials to have a final recommendation.

## Data Availability Statement

The raw data supporting the conclusions of this article will be made available by the authors, without undue reservation.

## Ethics Statement

The study was approved by the Online Studies Scientific Steering Committee of the Turkish Neonatal Society and by the Institutional Review Board of Ankara University. Written informed consent was obtained from the parents or guardians of the newborns.

## Author Contributions

EO and OE contributed substantially to article conception and design and drafted the manuscript. ZA, ND, HK, IG, SE, MC, GB, FO, HS, YC, HO, NK, BA, MT, KC, DA, AB, KT, MO, FT, and EE participated in acquisition of data. SA critically revised it. All authors gave their final approval to this manuscript and agree to be accountable for all aspects of the work, ensuring integrity and accuracy.

## Conflict of Interest

The authors declare that the research was conducted in the absence of any commercial or financial relationships that could be construed as a potential conflict of interest.
